# Historical Trends in Sexual Activity and Sexual Health in France (1992–2023): A Gendered Perspective

**DOI:** 10.1111/psrh.70078

**Published:** 2026-08-27

**Authors:** Nathalie Bajos, Michel Bozon, Rim Bellamine, Armelle Andro

**Affiliations:** ^1^ INSERM Paris France; ^2^ EHESS Paris France; ^3^ INED Aubervilliers France; ^4^ Paris 1 Pantheon‐Sorbonne University Paris France

**Keywords:** France, gender, historical trends, sexual behaviors, sexual health, activité sexuelle, France, genre, santé sexuelle, tendances historiques

## Abstract

**Objective:**

To analyze historical gender trends in sexual indicators in France over a period of three decades.

**Methods:**

We used data from three national probability surveys on sexual behaviors in France conducted in 1992 (*N* = 20,055), 2006 (*N* = 12,364), and 2023 (*N* = 19,015) to compare sexual indicators among men and women aged 18–69 years.

**Results:**

The median age at first sexual intercourse has increased since the late 2010s, reaching 18.2 years for women and 17.7 years for men in 2023. The average number of lifetime sexual partners for those who have already had a sexual partner increased from 3.4 in 1992, to 4.5 in 2006, to 8.0 in 2023 for women and from 11.2 in 1992, to 11.9 in 2006, and to 16.6 in 2023 for men. Meeting sexual partners online has become widespread, with 44% of women and 48% of men aged 18–29 doing so in 2023. The prevalence of same‐sex partners also increased, from 3% in 1992 to 4% in 2006 to 10% in 2023 for women and from 4% in 1992 and 2006 to 9% in 2023 for men. Sexual activity in the past year and the frequency of sexual intercourse in the last 4 weeks decreased among single respondents since the 2000s. Meanwhile, having multiple partners and having masturbated increased, especially among women.

**Conclusion:**

These changes, which reflect increases in women's sexual agency and may partly be attributed to the digital revolution, need to be considered when tailoring public health messages to these new realities.

## RÉSUMÉ

**Objectif:**

Analyzer les tendances historiques des indicateurs sexuels en France sur trois décennies dans une perspective genrée.

**Méthodes:**

Les données de trois enquêtes probabilistes nationales sur les comportements sexuels en France menées en 1992 (*n* = 20,055), 2006 (*n* = 12,364) et 2023 (*n* = 19,015) ont été utilisées pour comparer les indicateurs sexuels chez les femmes et les hommes âgés de 18 à 69 ans.

**Résultats:**

L'âge médian du premier rapport sexuel a augmenté depuis la fin des années 2010, atteignant 18.2 ans pour les femmes et 17.7 ans pour les hommes en 2023. Le nombre de partenaires sexuels au cours de la vie pour les personnes ayant déjà eu un partenaire sexuel est passé de 3.4 en 1992 à 4.5 en 2006 et 8.0 en 2023 pour les femmes et de 11.2 en 1992, 11.9 en 2006 et 16.6 en 2023 pour les hommes. La rencontre de partenaires sexuels en ligne s'est généralisée, 44% des femmes et 48% des hommes âgés de 18 à 29 ans l'ayant fait en 2023. La fréquence des rapports avec des partenaires de même sexe a augmenté, passant de 3% en 1992 à 4% en 2006, pour atteindre 10% en 2023 pour les femmes; et de 4% en 1992 et 2006, pour atteindre 9% en 2023 pour les hommes. L'activité sexuelle au cours de l'année écoulée et la fréquence des rapports sexuels au cours des quatre dernières semaines ont diminué chez les personnes célibataires depuis les années 2000. En revanche, le nombre de partenaires multiples et la masturbation ont augmenté, en particulier chez les femmes.

**Conclusion:**

Ces changements, qui peuvent être en partie attribués à la révolution numérique et qui reflètent aussi une plus grande autonomie des femmes, doivent être pris en compte pour adapter les messages de santé publique à ces nouvelles réalités.

## 1 Introduction

Analyzing trends in sexual activity over the last few decades in light of social, political, and epidemiological changes is essential to designing effective sexual health policies responsive to the social reality [1]. Historical analyses are as critical in informing public health policies as cross‐national comparisons [2]. Comparisons over time require repeated surveys conducted under consistent protocols to avoid the risk of being unable to determine whether observed differences are real or stem from methodological discrepancies [3, 4, 5, 6].

Recent studies investigating time trends in sexual activity in the United States (US) [7], Finland [8], Germany [9], and Japan [10] report a decline in sexual activity among younger cohorts. Similar decreases in sexual frequency among married or partnered individuals were noted in the US [7], Australia [11], and Britain [12].

These trends have emerged within a rapidly evolving society marked by changes in social life such as family composition, economic and health crises, digital advancements, and progress in gender equity and sexual rights. Digital technology has broadened sexual experiences to include virtual encounters while simultaneously accelerating the pace of modern life, intensifying stress from constantly balancing work, family responsibilities, and leisure [13]. Concurrently, the agenda for gender equity and sexual rights, through the denunciation of sexual harassment and gender‐based violence, has accelerated since the #MeToo social movement and the advancement of women's social status, alongside the legalization of same‐sex marriage, has reshaped normative expectations about sexual consent and relationships [14]. While current declines in sexual activity may be tied to these major social movements and shifting gender dynamics, indicators of presence and frequency of sexual activity in the last 12 months alone do not fully capture the evolving landscape of sexuality, from issues of consent to the diversification of sexual practices, further expanded by the rise of virtual sexual experiences.

To take stock of these significant changes, we draw on three large nationally representative surveys on sexuality in France conducted in 1992 [15] and 2006 [16], and 2023 (see Moreau and colleagues, this Special Issue, presenting the 2023's survey methodology in details), which use a common protocol to explore three fundamental components of sexuality: sexual practices, sexual relationships, and sexual norms. The objectives of each survey have evolved to address contemporary issues in sexual health: the 1992 survey focused on risky sexual behaviors related to HIV/AIDS during the early years of the pandemic, the 2006 survey examined the social and gendered contexts of sexuality, highlighting the diversification of attitudes and sexual practices, and the 2023 survey employed an intersectional approach to analyze inequalities in sexuality and sexual health throughout the life course. Despite evolving objectives, the common design and measurement of indicators across the three surveys allow a unique perspective on time trends of emerging adult sexuality and sexual practices over the life course, with a particular focus on gender and generational shifts.

## 2 Methods

### 2.1 Study Population

This analysis was based on three national population‐based probability surveys on sexual behaviors in France spanning a period of 30 years. The ACSF‐1992 survey included 20,055 participants aged 18–69 years, randomly selected from the phone directory, with over‐representation of men who had sex with men [15]. CSF‐2006 also comprised a national probability sample of 12,364 participants aged 18–69, selected using random digit dialing, with over‐representation of young adults [16]. Finally, CSF‐2023 expanded the sample to 19,015 individuals aged 18–69 years selected using the same process as the 2006 survey, with under‐representation of participants over 64 (see Moreau and colleagues, this Special Issue). All surveys were conducted by interviewers specially trained in issues relating to sexuality and sexual health. In this article, we included respondents aged 18–69 across the three samples to ensure age consistency.

We present the main methodological features of the three surveys in Table S1. The three studies were approved by the French National Commission for Information Technology and Civil Liberties (CNIL) and INSERM Ethical Review Board. All participants provided initial oral consent to participate and had access to written information about the study, by letter or mail, and on a dedicated website for the 2023 survey.

### 2.2 Measures

We considered the following indicators to characterize trends in sexual debut, lifetime sexual experiences, and recent sexual activity across the three surveys. Specifically, we used three indicators related to sexual debut: median age at first sexual intercourse, contraceptive use at first sexual intercourse, and external condom use at first sexual intercourse. We captured lifetime sexual experiences across several indicators including lifetime number of sexual partners and same‐sex partners, lifetime sexual practices including solo masturbation, oral sex, and anal sex, desired sexual intercourse, and history of sexual violence. Recent sexual activity referred to sexual intercourse in the prior year, frequency of sexual intercourse in the last 4 weeks, and current sexual satisfaction. Finally, we included one attitudinal measure comparing male versus female sexual needs.

Some questions were formulated slightly differently between the surveys. The question on frequency of sexual intercourse in the last 4 weeks referred to any acts in the 1992 and 2023 surveys, but only to acts with the last partner in the 2006 survey. Additionally, questions on sexual needs and desired sexual relations were not available in the 1992 survey. Finally, the question on sexual violence referred only to rape, without mentioning attempted rape, in the 1992 survey while the 2006 and 2023 surveys included both rape and attempted rape questions. Given the important differences in wording, we do not present data from the 1992 survey for this domain.

### 2.3 Statistical Analysis

In this paper, we compared sexuality indicators by gender and age across the three surveys for respondents aged 18–69 years old. We took into consideration differences in sampling design and response rates between surveys by weighting the data to adjust for unequal probabilities of selection, i.e., the number of eligible individuals in the household and age of the respondent. Additionally, we accounted for nonresponse bias by using similar post stratification weighting procedures for the three surveys, matching the distribution of sample characteristics by gender, age, level of education, region, and household composition with the distribution of the French population based on census data for each survey year [17] (see Moreau and colleagues, this Special Issue). This approach ensured that each survey provided representative estimates of the French population and allowed for valid comparisons across results.

We evaluated variations in sexuality indicators across gender, age groups, and over time based on the comparison of confidence intervals with additional statistical tests, which account for the complex sampling design and post‐stratification weights. We highlighted differences when the 95% confidence intervals did not overlap. Numbers reported in the tables are unweighted, i.e., the number of people who answered the questions, while all percentages are weighted. We conducted all analyses with the R software version R‐4.4.1.

## 3 Results

### 3.1 Timing of and Contraception and External Condom Use at First Sex

Our results indicate that over the last few decades the median age at first intercourse decreased by almost 3 years for women between the early 1960s and the middle of the 2000s (20.1 vs 17.3) and 1 year and a half for men (18.8 vs. 17.3). Consequently, the gender age gap at sexual debut decreased during this period (Figure 1). Since the end of the 2010s, trends have slightly reversed with an increase in the median age at first sex for both genders (18.2 for women and 17.7 for men in 2019–2023). However, these trends were not reflected in the percentage of respondents who initiated sex before age 15, which remained stable between 1992 and 2006 (3% and 3% of women, and 10% and 9% of men, respectively) and slightly increased to 7% for women and 12% for men in 2023 (Table S2).

**FIGURE 1:**
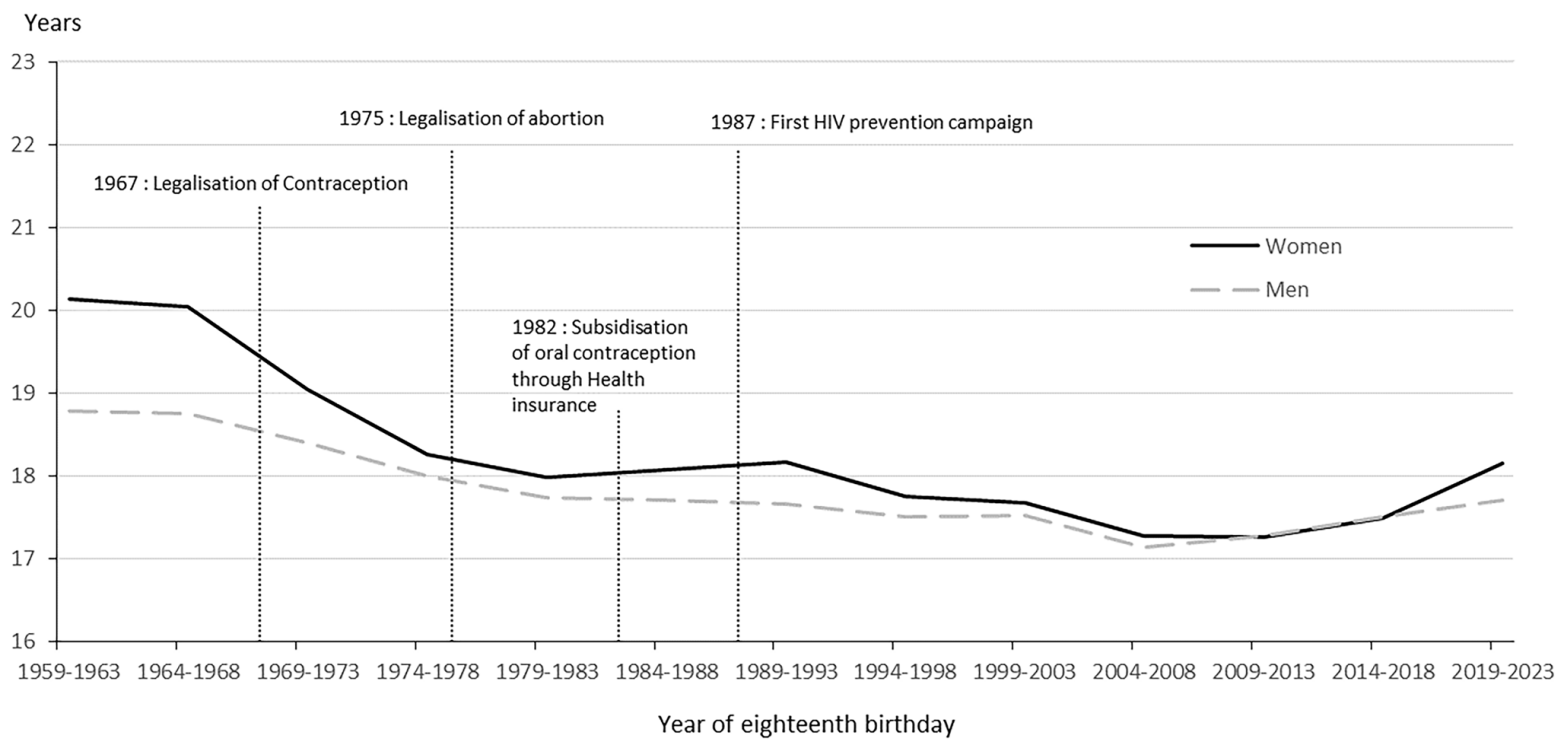
Median age at first sexual intercourse, by sex and age and year of the 18's birthday, among survey participants set against significant policy milestones in France.

The data also showed a significant increase in the use of any contraception at first sexual intercourse from the early 1960s to the middle of the 2000s (Figure 2) rising steadily from 23% in 1959 to 1963, before the legalization in 1967, to 96% in 2004–2006. After reaching its peak, contraceptive use at first sex declined to 83% among those who initiated sex between 2019 and 2023. We saw similar patterns for external condom use at first sex (Figure 2), although external condom use rose most strikingly during the 1980s, when prevention campaigns were launched to tackle the spread of HIV. External condom use at first sex decreased to 72% for women and 82% for men in the most recent period.

**FIGURE 2:**
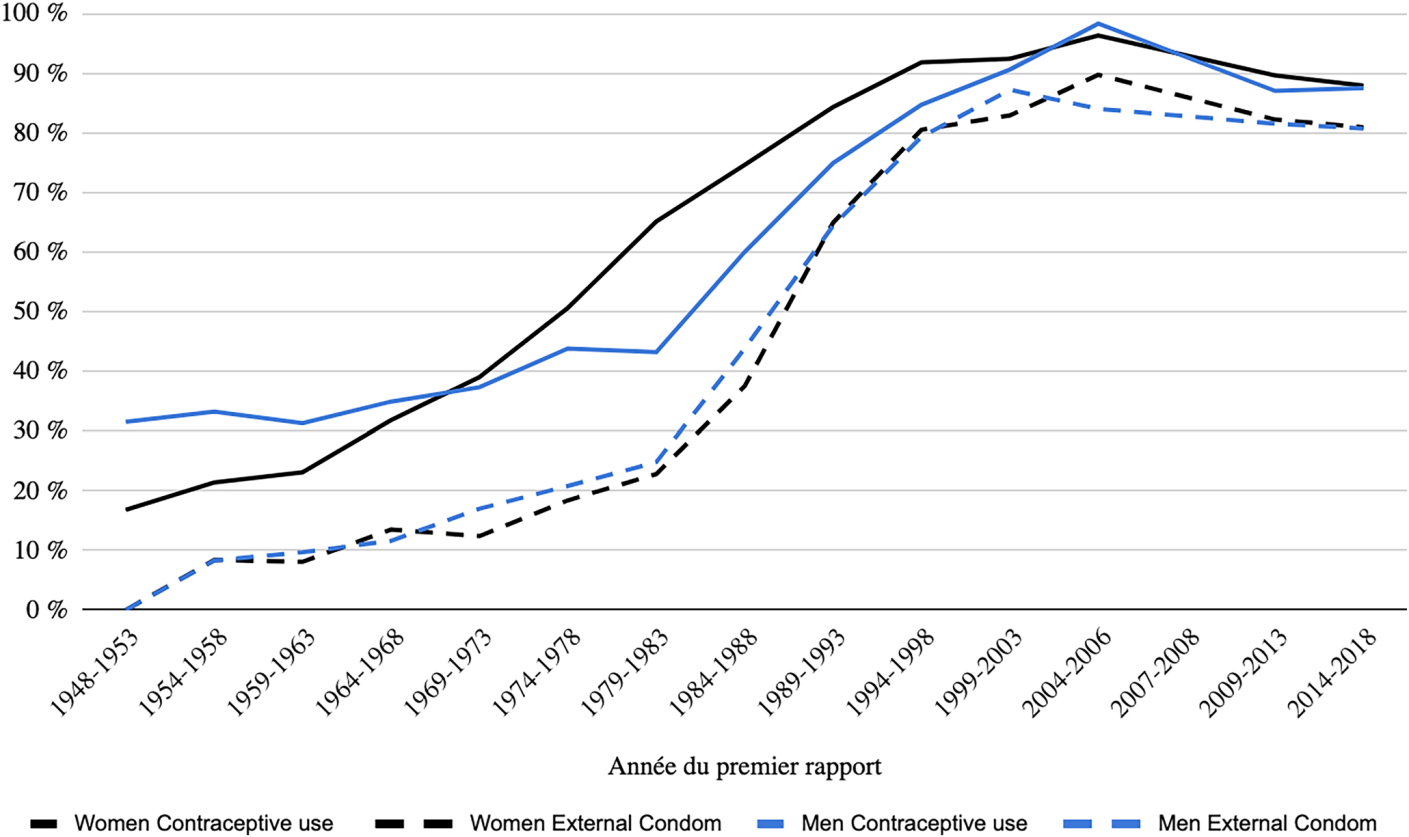
Use of contraception (all methods) at first heterosexual intercourse and external condom use for all among French survey participants.

### 3.2 Sexual Partnerships

Mean number of lifetime sexual partners among sexually active women increased over time, from 3.4 in 1992 to 4.5 in 2006 to 8.0 in 2023 (Table 1). The corresponding figures for men showed stability between 1992 and 2006 (11.2–11.9, respectively) and a substantial increase to 16.4 in 2023. [Correction added on 17 October 2026, after first online publication: In the previous sentence, 16.6 was changed to 16.4.] The mean number of lifetime partners increased across all age groups. [Correction added on 17 October 2026, after first online publication: Data in Table 1 was corrected and as a result two sentences were removed from the end of this paragraph due to a reinterpretation of the data.]

**TABLE 1:** Mean number of lifetime sexual partners,^a^ by sex and age (95% CI) among those who have had a sexual partner among survey participants in France across three decades. [Correction added on 17 October 2026, after first online publication: In Table 1, all data in the final row was corrected due to an error of transcription]

Age	Women			Men		
	1992	2006	2023	1992	2006	2023
18–29	4.1 (3.9–4.3)	4.0 (3.8–4.3)	7.3 (6.7–7.8)	10.5 (9.8–11)	7.8 (7.2–8.5)	11.3 (10–13)
30–39	4.4 (4.2–4.7)	5.2 (4.9–5.4)	9.9 (9.2–10.7)	12.4 (12–13)	12.1 (11–13)	17.2 (15–19)
40–49	3.3 (3.0–3.5)	5.2 (4.4–5.9)	8.9 (8.3–9.6)	11.8 (11–13)	14.2 (12–16)	17.7 (16–19)
50–59	2.3 (2.1–2.4)	4.3 (3.6–5.0)	7.5 (6.9–8.1)	10.7 (9.5–12)	13.8 (12–16)	18.0 (17–19)
60–69	1.8 (1.7–2.0)	3.2 (2.4–4.1)	6.3 (5.5–7.1)	9.6 (8.3–11)	11.8 (9.4–14)	17.7 (16–20)
18–69	3.4 (3.3–3.5)	4.5 (4.3–4.7)	8.0 (7.7–8.3)	11.2 (11–12)	11.9 (11–13)	16.4 (16–17)

^a^ The maximum number was capped at 200.

The formation of sexual relationships has also seen dramatic increases in the percentage of respondents who met their partners online. While 3% of women and 5% of men in 2006 reported that they had sex with a partner they had met online, this percentage rose to 25% for women and 32% for men in 2023 (Table S3). Close to half of women and men aged 18–29 (44% of women and 48% of men) had done so in 2023.

Sexual relations with same‐sex partners also increased over time, especially in the most recent period and for women. In 1992, 3% of women reported ever having a same‐sex partner. This increased to 4% in 2006 and to 10% in 2023. Among men, these percentages were 4%, 4%, and 9%, respectively (Table 2). For the first time in 2023, women were more likely to report same‐sex partners than men. Most respondents who reported same‐sex relationships also reported sexual relations with partners of the other gender. This trend was notable among young people 18–29 years: 16% of young women reported having sex with people of both genders and 1% with women only, compared with 5% and 0% in 2006 and 2% and 0% in 1992 respectively. Among young men of the same age, the changes were also significant (7% and 3% in 2023, 3% and 1% in 2006 and 3% and 0% in 1992).

**TABLE 2:** Proportion (%) of those who have had same‐sex partners over lifetime among those who have had a sexual partner, by sex and age (95% CI), among survey participants in France across three decades.

Age	Women						Men					
	1992		2006		2023		1992		2006		2023	
	Only same sex	Both sex	Only same sex	Both sex	Only same sex	Both sex	Only same sex	Both sex	Only same sex	Both sex	Only same sex	Both sex
18–29	0.0 (0.0–0.24)	1.9 (1.2–3.2)	0.0 (0.00–0.00)	5.4 (4.3–6.8)	1.3 (0.80–2.0)	16.0 (14–18)	0.2 (0.10–0.41)	2.5 (1.7–3.6)	0.7 (0.37–1.3)	3.1 (2.2–4.2)	3.2 (2.3–4.4)	7.4 (6.0–9.1)
30–39	0.0 (0.01–0.28)	3.8 (2.4–5.9)	0.2 (0.08–0.66)	5.5 (4.6–6.5)	0.5 (0.22–1.0)	14.1 (12–16)	0.2 (0.11–0.41)	4.2 (3.0–6.0)	0.2 (0.11–0.54)	5.5 (4.6–6.6)	1.9 (1.3–2.7)	7.2 (5.8–8.9)
40–49	0.1 (0.02–0.30)	2.0 (1.2–3.4)	0.0 (0.00–0.00)	3.9 (2.7–5.4)	0.0 (0.00–0.13)	9.3 (7.8–11)	0.0 (0.0–0.22)	5.4 (2.7–10)	0.2 (0.05–1.1)	3.9 (2.7–5.6)	1.1 (0.77–1.7)	7.0 (5.6–8.7)
50–59	0.0 (0.00–0.00)	3.9 (0.84–16)	0.0 (0.00–0.00)	3.0 (2.2–4.3)	0.3 (0.04–1.6)	6.3 (5.1–7.6)	0.2 (0.02–1.2)	3.2 (1.5–7.0)	0.0 (0.00–0.0)	3.1 (2.0–4.9)	1.0 (0.62–1.7)	6.7 (5.5–8.2)
60–69	0.0 (0.00–0.00)	0.9 (0.15–5.4)	0.0 (0.00–0.00)	1.1 (0.56–2.2)	0.1 (0.02–0.22)	3.5 (2.5–4.8)	0.0 (0.0–0.0)	3.0 (1.2–7.6)	0.0 (0.0–0.0)	2.8 (1.5–4.9)	0.5 (0.20–1.4)	6.5 (5.1–8.3)
18–69	0.0 (0.01–0.09)	2.5 (1.6–3.9)	0.1 (0.02–0.15)	4.0 (3.5–4.5)	0.4 (0.26–0.61)	9.6 (8.9–10)	0.1 (0.08–0.23)	3.7 (2.8–4.9)	0.3 (0.15–0.42)	3.8 (3.2–4.4)	1.5 (1.3–1.9)	7.0 (6.3–7.7)

### 3.3 Sexual Activity in the Last 12 Months and Last 4 Weeks

The percentage of respondents who had sexual intercourse in the last 12 months decreased over time, for both genders and in all age groups (Table 3). In 1992, 86% of women aged 18–69 had sexual intercourse in the past year, and this proportion dropped to 83% in 2006 and 78% in 2023. Similarly, the percentage of men reporting sexual intercourse in the past year fell from 92% in 1992 to 89% in 2006 to 82% in 2023. The decline was particularly pronounced for single people, but less marked for couples and insignificant between 2006 and 2023 when broken down by age groups (data not shown). The decline was partly due to delayed sexual debut, as the decline was less pronounced among respondents who ever had sex (Table S4).

**TABLE 3:** Proportion (%) of having sexual intercourse^a^ and having several partners in the last 12 months^b^ (> 1 part), by sex and age (95% CI) among survey participants in France across three decades.

Age	Women						Men					
	1992		2006		2023		1992		2006		2023	
	SI‐12	> 1 part	SI‐12	> 1 part	SI‐12	> 1 part	SI‐12	> 1 part	SI‐12	> 1 part	SI‐12	> 1 part
18–29	83.7 (82–85)	9.6 (8.6–11)	85.5 (83–87)	19.3 (17–21)	79.6 (77–82)	23.9 (22–26)	85.9 (84–87)	22.9 (21–25)	85.3 (83–87)	29.0 (27–31)	74.2 (72–77)	32.3 (30–35)
30–39	96.6 (96–97)	5.0 (4.4–5.7)	93.5 (92–95)	6.9 (6.1–7.9)	91.1 (89–93)	11.0 (9.3–13)	97.0 (96–98)	9.7 (8.8–11)	91.4 (90–93)	10.9 (9.6–12)	89.2 (87–91)	16.6 (14–19)
40–49	93.5 (92–94)	3.8 (3.2–4.6)	89.5 (87–92)	5.6 (4.4–7.1)	85.4 (83–87)	8.3 (6.8–10)	97.3 (96–98)	8.9 (7.9–10)	92.4 (90–94)	7.3 (5.8–9.2)	89.1 (87–91)	13.1 (11–15)
50–59	83.0 (81–85)	1.9 (1.4–2.5)	78.9 (76–82)	1.7 (1.2–2.5)	74.0 (71–76)	3.0 (2.3–3.9)	95.1 (94–96)	4.5 (3.6–5.6)	91.2 (88–93)	8.1 (6.4–10)	80.8 (78–83)	11.5 (9.8–14)
60–69	66.6 (64–69)	0.3 (0.15–0.78)	59.0 (55–63)	1.2 (0.66–2.1)	59.1 (56–62)	1.5 (0.87–2.5)	85.1 (83–87)	3.2 (2.4–4.1)	83.9 (80–87)	4.1 (2.8–6.0)	78.2 (75–81)	6.9 (5.5–8.8)
18–69	86.4 (86–87)	4.9 (4.5–5.3)	82.9 (82–84)	7.3 (6.7–7.9)	77.7 (77–79)	9.1 (8.5–9.9)	92.1 (91–93)	11.6 (11–12)	89.1 (88–90)	12.6 (12–14)	82.1 (81–83)	16.0 (15–17)

^a^ All participants.

^b^ Having had in sexual partner lifetime.

The frequency of sexual intercourse in the last 4 weeks among respondents who reported having had sexual intercourse in the last year dropped in all age groups between 2006 and 2023 (Table 4). Women who had sex in the last 12 months had on average 8.1 (7.5–8.7) acts of sexual intercourse (vaginal and/or anal penetration) in the last 4 weeks in 1992, 8.6 (8.4–8.8) in 2006, and dropped to 6.0 (5.8–6.2) in 2023. For men, the figures were 9.0 (8.5–9.5) in 1992, 8.7 (8.4–9.0) in 2006, and 6.7 (6.5–7.0) in 2023. We also observe this decline if we only consider cohabiting respondents and those who only had one partner in the last year (Table S5).

**TABLE 4:** Mean number of sexual intercourses in past 4 weeks^a^ in 1992, 2006, and 2023 (95% CI) among survey participants in France.

Age	Women			Men		
	1992	2006	2023	1992	2006	2023
18–29	10.1 (8.8–11)	11.0 (11–12)	7.9 (7.4–8.3)	11.1 (10–12)	12.3 (12–13)	9.1 (8.2–9.9)
30–39	9.3 (8.5–10)	9.5 (9.2–9.8)	6.9 (6.5–7.4)	10.4 (9.6–11)	9.4 (9.0–9.7)	7.7 (7.1–8.2)
40–49	9.1 (8.1–10)	8.5 (8.0–9.0)	6.2 (5.9–6.6)	9.9 (8.7–11)	8.8 (8.0–9.6)	7.1 (6.5–7.6)
50–59	5.3 (4.3–6.3)	7.0 (6.4–7.5)	5.0 (4.7–5.3)	6.9 (5.7–8.2)	6.9 (6.4–7.4)	5.8 (5.4–6.2)
60–69	4.4 (2.0–6.8)	5.2 (4.7–5.6)	3.3 (3.1–3.6)	5.4 (4.1–6.7)	5.7 (5.1–6.3)	4.3 (3.9–4.6)
18–69	8.1 (7.5–8.7)	8.6 (8.4–8.8)	6.0 (5.8–6.2)	9.0 (8.5–9.5)	8.7 (8.4–9.0)	6.7 (6.5–7.0)

*Note:* Number of sexual intercourses in the last month with the last partner in 2006, in general for 1992 and 2023 among those who had sexual intercourse in the last 12 months and who live in a couple or who reported having a so‐called “privileged partner.”

^a^ The maximum number was capped at 100.

While the frequency of sexual intercourse in the last 4 weeks decreased, among those who have already had a sexual partner, the percentage of respondents who had more than one partner increased, especially among young people 18–29 years old (10% in 1992, 19% in 2006, and 24% in 2023 for women; 23% in 1992, 29% in 2006, and 32% in 2023 for their male counterparts) (Table S4). As such, the gender gap has narrowed over time.

The range of sexual practices also diversified over time, with increasing percentages of men and women reporting sexual practices other than penile–vaginal intercourse (such as masturbation, oral sex, and anal intercourse) over time (Table 5). The increase in reported masturbation occurred across all age groups and was much more pronounced for women than for men. In 1992, 42% of women reported ever practicing masturbation, a figure that rose to 57% in 2006 and 73% in 2023. For men, the increase was more modest, from 83% in 1992 to 90% in 2006 and 93% in 2023. Experience of fellatio among those who had already had a sexual partner also increased over time, from 63% in 1992 to 78% in 2006 to 84% in 2023 among women and from 75% to 86% and 91% among men. Similar trends were observed for the experience of cunnilingus: from 72% in 1992 to 84% in 2006 to 87% in 2023 among women, and from 78% to 86% to 88% among men. Anal penetration also increased over time, rising from 23% in 1992 to 35% in 2006 to 39% in 2023 among women, and from 30% to 46% to 57% among men. This practice tended to be integrated later into the sexual lifespan, being more common among individuals aged 30–39 than those aged 18–29.

**TABLE 5:** Lifetime sexual practices (%) by sex and age; 1992, 2006, and 2023 (95% CI) among survey participants in France.

Age	Women			Men		
	1992	2006	2023	1992	2006	2023
Masturbation^a^						
18–29	42.8 (39–47)	55.9 (53–59)	75.6 (73–78)	85.4 (82–88)	92.5 (90–94)	94.0 (92–95)
30–39	63.3 (58–68)	65.7 (63–68)	77.6 (75–80)	83.5 (79–87)	92.3 (91–94)	94.5 (93–96)
40–49	44.2 (38–51)	61.6 (58–65)	77.6 (75–80)	82.2 (76–87)	91.8 (89–94)	94.6 (93–96)
50–59	22.0 (16–30)	55.2 (51–59)	71.0 (68–74)	83.5 (76–89)	87.5 (84–90)	90.9 (89–93)
60–69	29.1 (20–40)	37.4 (34–41)	63.1 (60–66)	76.4 (67–84)	82.2 (77–86)	89.0 (86–91)
18–69	42.4 (39–45)	56.5 (55–58)	72.9 (72–74)	82.8 (80–85)	89.9 (89–91)	92.6 (92–93)
Fellatio^b^						
18–29	65.6 (61–70)	80.0 (77–82)	86.6 (84–89)	78.9 (75–83)	86.2 (84–88)	92.1 (90–94)
30–39	81.8 (77–86)	86.6 (85–88)	88.7 (87–91)	84.1 (80–88)	92.0 (90–93)	93.8 (92–95)
40–49	69.9 (63–76)	83.1 (80–86)	87.1 (85–89)	77.4 (71–83)	90.0 (87–92)	92.7 (91–94)
50–59	38.4 (29–48)	75.3 (72–79)	83.0 (81–85)	65.9 (56–75)	82.2 (78–86)	89.1 (87–91)
60–69	47.9 (37–59)	60.2 (56–64)	77.3 (74–80)	60.6 (50–70)	71.6 (66–76)	85.0 (82–87)
18–69	63.2 (60–66)	78.3 (77–80)	84.4 (83–85)	75.3 (72–78)	85.5 (84–87)	90.5 (90–91)
Cunnilingus^b^						
18–29	74.4 (70–78)	86.5 (84–89)	89.2 (87–91)	77.5 (73–81)	83.2 (80–86)	83.9 (82–86)
30–39	87.9 (84–91)	90.8 (89–92)	90.3 (88–92)	88.7 (85–92)	89.8 (88–92)	88.4 (86–90)
40–49	77.4 (71–83)	88.4 (85–91)	89.1 (87–91)	84.0 (78–89)	91.8 (89–94)	89.8 (88–92)
50–59	53.2 (43–63)	80.4 (77–83)	85.8 (84–88)	65.6 (56–74)	85.0 (81–88)	88.2 (86–90)
60–69	56.7 (45–67)	66.3 (62–70)	80.5 (78–83)	64.0 (54–73)	74.9 (70–79)	88.1 (86–90)
18–69	72.1 (69–75)	83.7 (82–85)	86.9 (86–88)	77.8 (75–80)	85.7 (84–87)	87.7 (87–89)
Anal sex^b^						
18–29	22.0 (19–26)	33.0 (30–36)	33.5 (30–35)	32.2 (28–36)	40.0 (37–43)	46.1 (43–49)
30–39	34.0 (29–39)	42.2 (40–45)	49.0 (46–52)	39.9 (35–45)	57.0 (54–60)	64.5 (61–67)
40–49	26.0 (21–32)	41.2 (37–45)	46.4 (44–49)	30.6 (25–37)	53.1 (49–57)	66.1 (63–69)
50–59	16.5 (10–25)	33.6 (30–37)	38.4 (36–41)	20.7 (15–28)	43.4 (39–48)	58.6 (56–61)
60–69	13.4 (7.4–23)	20.8 (18–24)	28.3 (26–31)	15.7 (10–24)	32.6 (28–38)	51.1 (48–54)
18–69	23.4 (21–26)	35.2 (34–37)	38.9 (38–40)	29.6 (27–32)	46.3 (45–48)	57.4 (56–59)

^a^ Practised by a partner or by the respondent.

^b^ All those having had a lifetime sexual partner.

### 3.4 Sexual Violence

Reports of sexual violence have risen sharply over time. The overall increase in reported cases of rape or attempted rape between 2006 and 2023 was significant, rising from 16% to 30% among women and from 5% to 9% among men (Table 6). This increase was particularly pronounced in the 18–29 age group, rising from 17% to 38% for women and from 5% to 13% for men. Comparison of indicators following the same generation across two cross‐sectional survey rounds suggests a reporting effect, as 19% of women aged 30–39 in 2006 reported sexual violence, while the corresponding figure was 27% for those aged 47–56 in 2023, who were 17 years older than in 2006 (data not shown).

**TABLE 6:** Lifetime sexual violence (%) by sex and age (95% CI) among 2006 and 2023 survey participants in France.

Age	Women				Men			
	2006		2023		2006		2023	
	Rape	Rape + attempted rape	Rape	Rape + attempted rape	Rape	Rape + attempted rape	Rape	Rape + attempted rape
18–29	6.6 (5.5–8.0)	16.5 (15–18)	25.8 (21.1–30.4)	37.6 (33.3–41.8)	1.9 (1.2–3.0)	4.7 (3.6–6.1)	7.0 (1.5–12.4)	12.6 (7.4–17.8)
30–39	8.2 (7.0–9.5)	19.5 (18–21)	22.7 (17.3–28.1)	34.6 (29.7–39.50)	1.7 (1.2–2.5)	4.8 (3.9–5.8)	6.3 (0.4–12.3)	10.0 (4.4–15.7)
40–49	9.1 (7.2–11)	17.7 (15–21)	20.4 (15.3–25.5)	30.5 (25.9–35.1)	2.1 (1.2–3.8)	6.2 (4.5–8.5)	4.9 (0.0–11.0)	8.7 (2.9–14.4)
50–59	5.4 (4.1–7.2)	14.3 (12–17)	16.1 (11.1–21.1)	26.9 (22.2–31.5)	0.8 (0.34–1.8)	2.8 (1.8–4.5)	5.0 (0.0–11.2)	8.1 (2.4–13.8)
60–69	3.2 (2.2–4.7)	9.1 (7.2–11)	13.3 (7.3–19.4)	21.8 (16.4–27.3)	1.3 (0.50–3.4)	3.9 (2.3–6.4)	2.4 (0.0–8.5)	5.4 (0.0–12.1)
18–69	6.8 (6.1–7.5)	15.9 (15–17)	19.6 (17.3–22.0)	30.3 (28.2–32.4)	1.6 (1.2–2.1)	4.6 (3.9–5.3)	5.2 (2.5–7.8)	9.0 (6.5–11.6)

### 3.5 Sexual Satisfaction

The proportion of respondents who had sex to “please their partner without really wanting to do so” fell since the mid‐2000s, particularly among women: from 51% in 2006 to 43% in 2023 compared to 24% in 2006 and 23% in 2023 for men (Figure S1). The proportion of women reporting they were very satisfied with their current sexual life has remained stable over time: 47% in 1992, 44% in 2006, and 45% in 2023. On the other hand, current sexual satisfaction has declined over time for men, from 45% in 1992 to 35% in 2006 to 39% in 2023 (Table S6).

## 4 Discussion

The results show significant changes emerging in adult sexuality and sexual practices over time, with a reduction in partnered sexual activity and a diversification of sexual practices and partnerships. Recent trends indicate delayed first sexual intercourse and a decrease in the frequency of sexual intercourse, while at the same time, sexual experiences have continued to diversify, with increases in nonpartnered and nonpenile–vaginal practices, numbers of partners, and same‐sex experiences. Declining sexual activity was especially pronounced for single people and young men while the diversification of sexual activity and partnerships was more pronounced for young women, reducing gender gaps in sexuality. Reports of sexual violence have also increased, and at the same time, women are less likely to engage in sexual activity with their partners when they do not want to.

The decline in the age of first sex has been observed in other high‐income countries, including the United Kingdom (UK) [18], Norway [19, 20], and the US [21]. Similarly, declines in sexual activity among 15‐year‐old students in multiple European countries have been reported between 2010 and 2018, with changing gender patterns in early sexual initiation between regions, favoring girls' earlier first sex in the North versus boys' earlier initiation in the East and Southern regions of Europe [22]. In our study, we note a general convergence of median ages at sexual debut between women and men over several years, although the COVID‐19 period seems to have further delayed age at first sexual intercourse for young women, increasing gender differences. Although restricted mobility during the pandemic may partly explain the recent delay in first sexual intercourse, the more pronounced delay among women could also indicate increased reflexivity toward sexuality, following the #MeToo movement's heightened focus on consent within social and political discourse. Regardless, gender convergence or divergence in the age at first sexual experience does not imply that this event is no longer gendered [23], as qualitative research on the subject also shows [24].

Delays in first sexual intercourse do not coincide with greater contraceptive or external condom use at first sexual intercourse. In fact, while contraceptive use and external condom use rose steadily until the mid‐2000s, the recent declines are worrisome and may contribute to the rising rates of sexually transmitted infections reported since 2018 [25]. While the 2012 oral contraceptive pill scare in France has precipitated a disaffection for hormonal contraception [26], declines in external condom use, especially for girls, raise questions about the reduced intensity of prevention campaigns in recent years in France.

Alongside delays in sexual initiation, sexual activity in the last 12 months among single people and the frequency of sexual intercourse in the last 4 weeks have declined since the mid‐2000s. This phenomenon seems common across high income settings, with declines in sexual activity noted in the US, Finland, the UK, and Japan. In the US, sexual inactivity in the last year rose between 2000 and 2018 for men 18–34 years and women 25–34 years while the weekly number of sexual acts declined among married men and women [7, 27]. Likewise, the proportion of young people 18–39 with no experience of heterosexual activity increased in Japan between 1987 and 2015, with greater inactivity among low income, unemployed, or temporary/part‐time employed men [10]. Declines were also observed in Germany, where men's sexual activity in the last year fell from 81% in 2005 to 73% in 2016 [9] as well as Australia between 2000 and 2010 [11] and Britain [12].

Analyzing sexual activity solely in terms of the number of acts of sexual intercourse over a given period reflects a heteronormative perspective that places great importance on penetration [28]. Indeed, these trends do not necessarily signal an absence of sexuality, as physical sexual practices have become more diversified, with emerging digital sexual exchanges as part of a major shift in dating scenarios and where an increasing proportion of the population is meeting sexual partners online. The major impact of the digital revolution goes well beyond online dating, affecting the type of sexual practice, as shown in the article on this topic in the same issue (see Andro and colleagues, this Special Issue, analyzing sexual practices in the digital space). The diversification of sexual experiences over time is particularly notable among women in the most recent period, as they report more sexual partners, bi‐sexual relations, solo masturbation, and nonvaginal sexual practices. Significant, but less pronounced changes are recorded among men, leading to a reduction in the gender gap, although men's sexual behaviors remain more diversified in 2023, with the exception of bi‐sexual relations. The diversification of sexual activities has been documented in the mid‐2000s in other high‐income countries such as Scandinavian countries [20, 29], Great Britain [18, 30], and Australia [11]. One might think that the changes observed in France between the 2006 and 2023 surveys partly reflect changes in practices linked to the impact of the COVID‐19 pandemic. We have no quantitative data in France to document this issue. However, according to a recent study conducted in Great Britain, which allows for a comparison of the situation before and after the lockdown period implemented in March 2020, the COVID‐19 epidemic did not profoundly affect people's sexual activity [31].

Together, these changing patterns of sexual behaviors should be considered within the context of evolving social models of relationships and cohabitation, which influence the occurrence of sexual intercourse [32, 33]. On the one hand, men and women under 69 years old are less likely to live as a couple today in France compared to previous decades [34]. On the other hand, over their lifetimes, men and women experience a greater number of significant romantic or couple relationships than previous generations [35]. Periods without a stable partner are therefore more common today than in the past. These trends should also be considered in light of evolving social norms regarding sexuality. Notably, the differentialist perspective on sexuality, which holds that men have inherently greater sexual needs than women [36], is no longer the majority view, regardless of age and gender (see Table S7), whereas it was in 2006 [16]. This normative change can lead to, as well as reflect, a lower frequency of undesired sexual encounters.

How can we explain what might be described as the “contemporary paradox of sexuality,” characterized by a greater number of sexual partners and a wider array of practices, yet a decline in the frequency of sexual intercourse over the past month? The widespread use of sex toys may also explain the decline in the number of sexual encounters in the traditional sense with a partner [37]. Furthermore, some authors have attributed the decline in sexual activity among middle‐aged women to tiredness resulting from contemporary midlife challenges [38], an argument that could be extended to individuals of both sexes whose professional lives are more precarious and stressful.

Obviously, detailed analyses are needed. However, our results already provide additional elements for interpretation. The responses do not indicate a diminished interest in sexuality, as evidenced by the stability of sexual satisfaction levels, the stability of sexual activity for people living in a couple, the widespread engagement in sexual exchanges in the digital space, the increasing number of sexual partners, and the increased practice of masturbation, especially among young women. The sharp increase in masturbation among women can be seen as a greater assertion of their sexual expectations [39].

Our results suggest that it is ultimately the contours of sexuality that are evolving, with traditional hetero‐penetrative sexuality gradually giving way to more diversified forms of sexual activities, sometimes taking place in the digital space, and more often desired. A recent qualitative study based on semistructured interviews in France shows that an increasing number of women are engaged in a process of learning or relearning desire [40] and are therefore more likely to avoid sexual encounters that they do not really want. This latter point is not inconsistent with the continuing increase in reports of sexual violence, a trend that began well before the #MeToo movement [41] and has also been recorded in other surveys in France [42] and other countries such as Ireland [43]. This increase is likely to reflect not so much a real rise in the occurrence of such events as a stronger social and political rejection of violence [16]. It nonetheless requires the full attention of scientists and public policy makers. These developments are likely to be related to the improved social status of women and greater reflexivity, particularly among women, about what is expected of their sexual lives.

## 5 Limitations

These results should be considered with several limitations. Changes in sexuality and sexual health over time may be due to changes in survey methodology. In France, as in many other countries, response rates in national surveys have decreased over time, regardless of the method of data collection [2]. Nevertheless, the bias in the structure of the samples that may result from refusals was accounted for by post‐stratification weights using the same methodology for the three samples. In addition, responses to a survey on sexuality and sexual health are sensitive to the social context in which they are asked [5]. Changes observed over time may be the result of changes in the meaning of the terms used to describe sexual activity, for example what people understand by sexual intercourse [1, 19, 44]. Ultimately, these changes may also reflect a social environment that has become more open regarding sexual matters, particularly in relation to the reporting of practices that were socially stigmatized in earlier decades, such as same‐sex relationships or female masturbation [14].

## 6 Conclusion

Reflecting on the historical trends in sexual practices offers a distinct opportunity to gain insights into the social dynamics altering sexual activities and to tailor sexual health policies to these changes.

## Funding

The surveys in 1992, 2006, and 2023 were funded by the French National Agency of Aids Research (ANRS). The Fondation de France, the Institut National de la Prevention et d'Education pour la Sante, and the Direction de la Recherche, de l'Evaluation, des Etudes et des Statistiques (DREES), Santé publique France (SpF), and the Mission Interministérielle de Lutte contre les Drogues et les Conduites Addictives (MILDECA) also contributed to the funding. This work was supported by the Institut national de la santé et de la recherche médicale (Inserm), France, through the COUPERIN CY26 consortium.

## Supporting information


**Table S1:** Methodological characteristics of the three national surveys of sexual behavior and sexual health in France.
**Table S2:** Proportion (%) of those who had their first intercourse before 15 years old, by sex and age (95% CI) (all people) among survey participants in France across three decades.
**Table S3:** Proportion (%) of those who met a sexual partner through internet, by sex and age among those who have had a sexual partner (95% CI) among survey participants in France across three decades.
**Table S4:** Proportion (%) of sexual intercourse in the last 12 months (SI‐12), and having several partners in the last 12 months (> 1 part), by sex and age among those who have had a sexual partner lifetime (95% CI) among survey participants in France across three decades.
**Table S5:** Mean number of sexual intercourses in past 4 weeks; 1992, 2006 and 2023 among those who live in a couple and had only one partner in the last 12 months (95% CI) among survey participants in France.
**Table S6:** Proportion (%) of those who are very satisfied of their actual sexual life (95% CI) (all population) among survey participants in France across three decades.
**Table S7:** Do you agree with the opinion that “By nature, men have more sexual needs than women”? (%) Responses of survey participants in France.
**Figure S1:** Having sex without really wanting to in order to please the partner (often or sometimes) by sex and age (%) among survey participants in France in 2006 and 2023.
