# Supplementary material for: Historical Trends in Sexual Activity and Sexual Health in France (1992–2023): A Gendered Perspective
Source: Perspect Sex Reprod Health. 2026 Aug 27;58(3):258–69. doi: 10.1111/psrh.70078 (PMC13727500; doi:10.1111/psrh.70078)
Supplement: Supplementary file 1 [file PSRH-58-258-s001.docx]

**Supplementary Table 1: Methodological characteristics of the 3 national surveys of sexual behaviour and sexual health in France**

|  | ACSF-1992 | CSF-2006 | CSF-2023 |
| --- | --- | --- | --- |
| General thematic | Sexual Behavior and HIV prevention | Social Context of Sexuality | Sexualities and sexual health |
| Sample size  (18-69 years old) | 20,046 (8,948 men, 11,098 women) | 12,364 (5,540 men, 6,824 women) | 19,015 (9,234 men, 9,781 women) |
| Sampling design | 2 phases random survey: household selected from the telephone directory, then individuals | 2 phases random survey: household selected from the telephone directory, then individuals  Additional random sample of individual with a mobile phone but no landline (n = 300) | 2 phases random survey: random mobile sample (80%) and household random sample (20%) with the individuals randomly selected |
| Length of the questionnaire | 45 mn | 49 mn | 35mn telephone + 30mn internet |
| Mode of administration | Telephone | Telephone | Telephone +internet |
| Post stratification | Age, gender, level  of education, region | Age, gender, level  of education, region | Age, gender, level of education, region |

**Supplementary Table 2: Proportion (%) of those who had their first intercourse before 15 years old, by sex and age (95% CI) (all people) among survey participants in France across three decades**

|  | Women | | | Men | | |
| --- | --- | --- | --- | --- | --- | --- |
| Age | 1992 | 2006 | 2023 | 1992 | 2006 | 2023 |
| 18-29 | 5.9 (4.1-8.4) | 5.3 (4.3-6.6) | 8.1 (6.7-9.8) | 11.9 (9.5-15) | 12.4 (11-14) | 15.0 (13-17) |
| 30-39 | 4.3 (2.4-7.4) | 3.9 (3.2-4.9) | 11.1 (9.4-13) | 10.8 (8.0-14) | 10.7 (9.2-12) | 15.1 (13-17) |
| 40-49 | 0.5 (0.15-2.0) | 2.4 (1.5-3.9) | 6.5 (5.2-8.1) | 10.0 (6.9-14) | 8.5 (6.5-11) | 11.0 (9.1-13) |
| 50-59 | 0. 0 (0.01-0.73) | 1.4 (0.83-2.5) | 5.1 (3.9-6.6) | 6.5 (3.3-12) | 6.4 (4.6-8.8) | 10.2 (8.4-12) |
| 60-69 | 0.0 (0.0-0.0) | 0.5 (0.13-1.6) | 2.6 (1.7-4.2) | 10.6 (5.8-19) | 4.8 (3.1-7.4) | 9.0 (7.1-11) |
| *18-69* | 2.7 (2.0-3.6) | 2.9 (2.5-3.4) | 6.7 (6.0-7.4) | 10.3 (8.7-12) | 9.0 (8.1-9.9) | 12.1 (11-13) |

**Supplementary Table 3: Proportion (%) of those who met a sexual partner through internet, by sex and age among those who have had a sexual partner (95% CI) among survey participants in France across three decades**

|  | Women |  | Men |  |
| --- | --- | --- | --- | --- |
| Age | 2006 | 2023 | 2006 | 2023 |
| 18-29 | 5.7 (4.5-7.2) | 43.5 (41-46) | 9.9 (8.1-12) | 48.4 (45-51) |
| 30-39 | 3.3 (2.6-4.2) | 36.9 (34-40) | 6.9 (5.6-8.5) | 45.4 (42-48) |
| 40-49 | 2.4 (1.5-4.0) | 24.2 (22-27) | 3.0 (1.9-4.8) | 32.2 (30-35) |
| 50-59 | 1.1 (0.60-2.0) | 14.6 (13-16) | 1.5 (0.75-2.8) | 21.4 (19-24) |
| 60-69 | 0.4 (0.13-1.3) | 7.9 (6.5-9.6) | 0.2 (0.03-1.7) | 12.7 (11-15) |
| *18-69* | *2.8 (2.3-3.2)* | *24.8 (24-26)* | *4.7 (4.1-5.4)* | *31.9 (31-33)* |

|  | **Supplementary Table 4: Proportion (%) of sexual intercourse in the last twelve months (SI-12), and having several partners in the last 12 months (>1 part), by sex and age among those who have had a sexual partner lifetime (95% CI) among survey participants in France across three decades** | | | | | | | | | | | | |
| --- | --- | --- | --- | --- | --- | --- | --- | --- | --- | --- | --- | --- | --- |
|  | Women | | | | | | Men | | | | | | |
|  | 1992 | | 2006 | | 2023 | | 1992 | | 2006 | | 2023 | | |
|  |  |  |  |  |  |  |  |  |  |  |  |  |  |
| Age | SI-12 | >1 part | SI-12 | >1 part | SI-12 | >1 part | SI-12 | >1 part | SI-12 | >1 part | SI-12 | >1 part |  |
| 18-29 | 96.6  (96-97) | 9.6  (8.6-11) | 95.3  (94-96) | 19.3  (17-21) | 93.3 (92-95) | 23.9  (22-26) | 95.4 (94-96) | 22.9 (21-25) | 92.9 (91-94) | 29.0 (27-31) | 86.8 (84-89) | 32.3  (30-35) |  |
| 30-39 | 97.1 (96-98) | 5.0  (4.4-5.7) | 94.2  (93-95) | 6.9  (6.1-7.9) | 92.5 (91-94) | 11.0 (9.3-13) | 97.8 (97-98) | 9.7  (8.8-11) | 93.1 (91-94) | 10.9 (9.6-12) | 91.5 (90-93) | 16.6  (14-19) |  |
| 40-49 | 94.2 (93-95) | 3.8  (3.2-4.6) | 90.0  (88-92) | 5.6  (4.4-7.1) | 85.9 (84-88) | 8.3  (6.8-10) | 98.1 (97-99) | 8.9  (7.9-10) | 93.2 (91-95) | 7.3  (5.8-9.2) | 90.1 (88-92) | 13.1  (11-15) |  |
| 50-59 | 84.6 (83-86) | 1.9  (1.4-2.5) | 79.0  (76-82) | 1.7  (1.2-2.5) | 74.4 (72-77) | 3.0  (2.3-3.9) | 95.7 (95-97) | 4.5  (3.6-5.6) | 91.9 (89-94) | 8.1  (6.4-10) | 82.0 (79-84) | 11.5 (9.8-14) |  |
| 60-69 | 68.2 (66-70) | 0.3  (0.15-0.78) | 59.4  (56-63) | 1.2  (0.66-2.1) | 59.8 (57-63) | 1.5 (0.87-2.5) | 86.7 (85-89) | 3.2  (2.4-4.1) | 84.1 (80-87) | 4.1  (2.8-6.0) | 78.8 (76-81) | 6.9  (5.5-8.8) |  |
| *18-69* | *90.6 (90-91)* | *4.9*  *(4.5-5.3)* | *85.2*  *(84-86)* | *7.3*  *(6.7-7.9)* | *80.7 (80-82)* | *9.1*  *(8.5-9.9)* | *95.6 (95-96)* | *11.6 (11-12)* | *92.2 (91-93)* | *12.6 (12-14)* | *85.8 (85-87)* | *16.0*  *(15-17*) |  |

**Supplementary Table 5: Mean number of sexual intercourses in past 4 weeks; 1992, 2006 and 2023 among those who live in a couple and had only one partner in the last 12 months (95% CI) among survey participants in France**

|  | Women | | | Men | | |
| --- | --- | --- | --- | --- | --- | --- |
| Age | 1992 | 2006 | 2023 | 1992 | 2006 | 2023 |
| 18-29 | 9.9 (8.6-11) | 10.6 (10-11) | 7.3 (6.8-7.8) | 10.4 (9.5-11) | 11.9 (11-13) | 8.4 (7.5-9.3) |
| 30-39 | 9.2 (8.5-10) | 9.3 (9.1-9.6) | 6.5 (6.0-6.9) | 10.2 (9.3-11) | 9.1 (8.8-9.4) | 7.1 (6.6-7.6) |
| 40-49 | 9.1 (8.0-10) | 8.5 (8.0-9.0) | 6.1 (5.7-6.5) | 9.7 (8.3-11) | 8.8 (8.0-9.6) | 6.5 (6.0-7.0) |
| 50-59 | 5.2 (4.2-6.3) | 6.9 (6.4-7.5) | 5.0 (4.6-5.3) | 6.6 (5.4-7.7) | 6.8 (6.3-7.4) | 5.4 (5.1-5.8) |
| 60-69 | 3.3 (2.1-4.4) | 5.2 (4.7-5.6 | 3.4 (3.1-3.7) | 5.3 (4.0-6.7) | 5.6 (5.0-6.1) | 4.3 (3.9-4.7) |
| *18-69* | *7.9 (7.4-8.5)* | *8.4 (8.2-8.6)* | *5.7 (5.5-5.9)* | *8.7 (8.2-9.2)* | *8.4 (8.1-8.7)* | *6.2 (6.0-6.4)* |

**Supplementary Table 6: Proportion (%) of those who are very satisfied of their actual sexual life (95% CI) (all population) among survey participants in France across three decades**

|  | Women | | | Men | | |
| --- | --- | --- | --- | --- | --- | --- |
| Age | 1992 | 2006 | 2023 | 1992 | 2006 | 2023 |
| 18-29 | 52.1 (48-57) | 47.6 (44-51) | 51.4 (49-54) | 46.9 (42-51) | 37.4 (34-41) | 42.3 (40-45) |
| 30-39 | 50.3 (45-56) | 51.3 (48-54) | 49.8 (47-53) | 48.1 (43-53) | 40.7 (37-44) | 43.4 (40-46) |
| 40-49 | 51.1 (44-58) | 46.3 (41-52) | 48.4 (46-51) | 49.5 (43-56) | 32.0 (27-37) | 42.6 (40-46) |
| 50-59 | 43.6 (34-54) | 34.7 (30-40) | 42.7 (40-46) | 37.3 (28-47) | 30.6 (26-36) | 38.1 (35-41) |
| 60-69 | 31.8 (22-43) | 33.7 (29-39) | 34.3 (31-38) | 37.7 (28-48) | 33.7 (28-40) | 28.0 (25-31) |
| *18-69* | *47.0 (44-50)* | *43.6 (42-46)* | *45.3 (44-47)* | *45.0 (42-48)* | *35.1 (33-37)* | *39.0 (38-40)* |

**Supplementary Table 7: Do you agree with the opinion that "By nature, men have more sexual needs than women"? (%) Responses of survey participants in France**

|  | Completely agree | | Partly agree | | Partly disagree | | Completely disagree | | Do not know | | Total |
| --- | --- | --- | --- | --- | --- | --- | --- | --- | --- | --- | --- |
| Age | Men | Women | Men | Women | Men | Women | Men | Women | Men | Women |  |
| 18-29 | 8.0 | 7.6 | 17.9 | 19.5 | 26.4 | 21.2 | 33.7 | 46.0 | 13.8 | 5.6 | 100 |
| 30-39 | 12.7 | 16.5 | 23.1 | 27.6 | 21.8 | 20.2 | 27.0 | 29.0 | 15.4 | 6.8 | 100 |
| 40-49 | 12.7 | 18.8 | 23.6 | 31.2 | 22.1 | 20.6 | 26.6 | 19.2 | 14.0 | 10.2 | 100 |
| 50-59 | 15.4 | 25.8 | 23.6 | 28.7 | 14.8 | 16.6 | 19.8 | 16.5 | 26.3 | 12.4 | 100 |
| 60-69 | 15.4 | 20.9 | 22.3 | 36.4 | 18.6 | 11.3 | 20.6 | 16.6 | 23.1 | 14.7 | 100 |
| *18-69* | *12.9* | *19.5* | *24.1* | *28* | *18.5* | *17.3* | *25.6* | *23.3* | *18.8* | *11.9* | *100* |

**Supplementary Figure 1: Having sex without really wanting to in order to please the partner (often or sometimes) by sex and age (%) among survey participants in France in 2006 and 2023**
